# Association between high mobility group box 1 protein and juvenile idiopathic arthritis: a prospective longitudinal study

**DOI:** 10.1186/s12969-021-00587-1

**Published:** 2021-07-12

**Authors:** Dan Xu, Yu Zhang, Zhi-Yong Zhang, Xue-Mei Tang

**Affiliations:** 1 Department of Rheumatology and Immunology, Children’s Hospital of Chongqing Medical University, 136, Zhongshan 2nd Road, Yuzhong District, Chongqing, 400014 People’s Republic of China; 2 Ministry of Education Key Laboratory of Child Development and Disorders, National Clinical Research Center for Child Health and Disorders, China International Science and Technology Cooperation Base of Child Development and Critical Disorders, Chongqing Key Laboratory of Child Infection and Immunity, Children’s Hospital of Chongqing Medical University, Chongqing, People’s Republic of China

**Keywords:** HMGB1 protein, Inflammation, Juvenile idiopathic arthritis, Pediatrics

## Abstract

**Objective:**

To analyze the levels of high mobility group box 1 (HMGB1) protein on different courses of juvenile idiopathic arthritis (JIA).

**Methods:**

In our prospective longitudinal study, children with JIA were included with their blood samples collected at the first visit, 1-month, 3-month, and 6-month follow-up, respectively. Samples were also collected from healthy controls and children with reactive arthritis at the first visit. Levels of HMGB1 were determined using enzyme-linked immunosorbent assays. Clinical disease characteristics and routine laboratory findings were analyzed as well.

**Results:**

A total of 64 children were enrolled, of whom 31 (48.4%) were female. The median age at the first visit for participants with JIA was 9.25 years (range, 1.42–15.42) and the median duration of disease was 2.38 months (range, 1.53–49.31). Serum HMGB1 levels at the first visit were significantly elevated in children with systemic JIA compared with other groups, and so were in enthesitis-related arthritis versus healthy controls. Significant correlations were established at the first visit between HMGB1 levels and duration of disease, C-reactive protein, percentage of neutrophils, and ferritin. Data from all samples revealed that serum HMGB1 levels in JIA were significantly associated with erythrocyte sedimentation rates, C-reactive protein, percentage of neutrophils, and disease activity scores.

**Conclusions:**

Serum HMGB1 may be associated with clinical disease activity of JIA and specifically increased at the first visit in children with systemic JIA, suggesting its function as a sensitive inflammatory marker. Further large-scale studies are warranted to explore its spectrum in JIA.

## Introduction

Juvenile idiopathic arthritis (JIA) has been considered as the most common rheumatic disease characterized by complex chronic inflammation of unknown cause in children, representing a heterogeneous group of disorders all sharing the clinical manifestation of arthritis [1]. The worldwide incidence rates per 100,000 range from 0.8 to 22.6 and the prevalence rates are from 7 to 401 per 100,000 [2, 3]. The International League of Associations for Rheumatology (ILAR) proposed a classification that divided JIA into 7 subgroups to standardize nomenclature in 1995, which has been widely adopted at present [4]. Most subgroups have two criteria in common: age at onset < 16 years and persistent arthritis for more than 6 weeks, while systemic JIA needs 2 weeks of fever and arthritis for diagnosis. The clinical courses among subgroups and individuals vary to a large degree, making it difficult to manage JIA in a standard fashion. Thus far, mechanisms involved in the pathogenesis of JIA and immunological profiles of different subgroups have not been completely understood. In this setting, novel biomarkers of inflammation and joint destruction are required to be developed so that the disease progression would be more precisely monitored. Besides, it is possible to discover new potential therapeutic targets and define several subgroups of JIA better.

High mobility group box 1 (HMGB1) protein, a nuclear DNA binding protein, has been recognized as a new pro-inflammatory cytokine on the extracellular functions, having the capacity to drive the pathogenesis of chronic inflammatory conditions including systemic lupus erythematosus and rheumatoid arthritis [5]. Through multiple receptors, HMGB1 released during the inflammatory phase can activate the NF-kB pathway and produce cytokines and chemokines for the inflammation and immune response [6]. Targeting HMGB1 directly or toward HMGB1-receptor signaling could be a promising approach in treating sepsis, arthritis, cancer, diabetes, and JIA [7, 8]. Previous studies have demonstrated that levels of HMGB1 in serum were increased in patients with autoimmune diseases and correlated with disease activity [9–11]. For children with JIA, females are more likely to be positive for anti-nuclear antibodies and HMGB1 antibodies [12]. It has been shown that HMGB1 levels were associated with inflammatory activity, early-onset, disease progression, and long-term prognosis in children with JIA [13, 14]. Reactive arthritis (ReA) refers to acute self-limited aseptic arthritis related to previous extra-articular infection that can follow streptococcal infection and genitourinary or/and gastrointestinal infection, which occasionally shares a similar clinical presentation with JIA. There is an absence of a consensus regarding ReA diagnostic criteria in the majority of cases and the diagnosis of ReA is established on the association of clinical and microbiological criteria [15]. To date, levels of HMGB1 on different courses of JIA subgroups and in the differential diagnosis have not been fully investigated. This prospective longitudinal study aimed to analyze the levels of HMGB1 in healthy controls, ReA, and different subgroups of JIA. Additionally, we assessed the changes in serum HMGB1 on different courses of JIA.

## Methods

### Patient population

The local institutional review board approved the protocol. Informed consent to participate in the study was provided by a parent or legal guardian before patient recruitment. Patients with JIA, ReA, and healthy controls, matched by sex and age, were randomly assigned in the Children’s Hospital of Chongqing Medical University and their blood samples were collected for laboratory studies. The inclusion criteria for JIA were as follows: age at first visit < 16 years, arthritis of unknown cause > 6 weeks (fever and arthritis for 2 weeks in systemic JIA), and new diagnosis without prior therapy. Patients were excluded if they were unable to provide complete information regarding arthritis, lost to follow-up, or diagnosed with macrophage activation syndrome within the last 6 months, tuberculosis, connective tissue disease, and other infections such as brucellosis, Lyme disease, etc. The diagnosis of ReA was mainly based on the history of extra-articular infection within 4 weeks and comprehensive physical examinations. Laboratory study or imaging findings may provide useful information for differential diagnosis.

### Clinical assessment and management

Medical records and clinical disease characteristics were analyzed in the cohort of JIA, including sex, age at first visit, duration of disease, number of the inflamed joints, associated complications, as well as laboratory and imaging findings. The laboratory examinations were performed at patients’ enrollment to determine C-reactive protein (CRP), erythrocyte sedimentation rate (ESR), differential white blood cells, ferritin, common families of interleukin (IL), tumor necrosis factor-α (TNF-α), interferon-γ (IFN-γ), rheumatoid factor (RF) and other autoantibodies. Levels of HMGB1 were evaluated using enzyme-linked immunosorbent assay (ELISA). X-rays, musculoskeletal ultrasonography, and magnetic resonance imaging (MRI) were taken into consideration to detect joints with active arthritis and exclude other conditions such as fractures, infection, tumors, or congenital defects. All patients with JIA were classified according to the ILAR revised criteria into the following categories: systemic JIA, oligoarticular JIA, RF-negative polyarticular JIA, RF-positive polyarticular JIA, enthesitis-related arthritis (ERA). Treatment strategies were tailored based on disease subgroup and severity, presence of poor prognostic indicators, and response to medications. The available drugs contained nonsteroidal anti-inflammatory drugs, glucocorticoids, conventional disease-modifying antirheumatic drugs, and biological agents such as infliximab and tocilizumab.

The Juvenile Arthritis Disease Activity Score (JADAS), more specifically JADAS-27, was used in our clinical practice to evaluate the disease activity of certain JIA subgroups (oligoarticular JIA, RF-negative polyarticular JIA, RF-positive polyarticular JIA), which consisted of four domains: physician global (0–10), patient or parent global (0–10), number of active joints, and normalized ESR [16]. The final result was aggregated by the points scored in each domain. According to the Wallace criteria, the clinically inactive disease was defined as the following: best possible score on physician global assessment of disease activity; no joints with active arthritis; no active uveitis; no fever, rash, serositis, splenomegaly or generalized lymphadenopathy attributable to JIA; ESR or CRP level within normal limits (high levels only acceptable if the cause is not JIA); and duration of morning stiffness for less than 15 min [17]. A cut-off JADAS of 2 in oligoarticular disease or 3.8 in polyarticular disease indicated minimal disease activity. Clinical remission was defined as JADAS≤1. Moreover, the Juvenile Spondyloarthritis Disease Activity Index (JSpADA) and the systemic Juvenile Arthritis Disease Activity Score (sJADAS) were used for ERA and systemic JIA, respectively, as they have been validated in children and shown improved performance in assessing the disease activity [18, 19].

Patients with JIA were routinely followed-up at 1, 3, and 6 months after the first admission, with a professional evaluation of disease activity by a pediatric rheumatologist. Clinical data were recorded at each visit on differential white blood cells, CRP, ESR, and HMGB1. Meanwhile, the JADAS-27, sJADAS-27, and JSpADA were calculated through collaboration among children, parents, and physicians.

### ELISA protocol for HMGB1

HMGB1 serum levels were quantitatively measured using an ELISA kit (IBL International GmbH, Hamburg, Germany) according to the manufacturer’s instructions about test procedures. Collected samples from JIA subgroups, ReA group, and healthy controls were centrifuged within 4 h at 2500×g for 10 min at 4 °C and frozen/stored at − 80 °C until used to perform the ELISA. Plates coated with anti-HMGB1 polyclonal antibody were incubated with serum samples for 22 h at 37 °C. Next, peroxidase-conjugated anti-HMGB1, 2-monoclonal antibody was applied as detection. The analytical sensitivity was 0.2 ng/mL in the highly sensitive range. Cross-reactivity with HMGB2 was less than 2% and below the lower limit of detection.

### Statistical analysis

Continuous variables were demonstrated as the median value (IQR: 25th, 75th) as appropriate. Categorical variables, presented as a percentage, were compared with the chi-square test or Fisher’s exact test. Comparisons of HMGB1 levels among subgroups were determined by the non-parametric Kruskal-Wallis test. Correlations between linearly related variables were assessed using a Spearman rank correlation with a coefficient (r_s_). Statistical analyses were performed using SAS® software, version 9.4 (SAS Institute, Cary, NC, USA). *P* values less than 0.05, two-sided, were considered to indicate statistical significance.

## Results

A total of 64 children were enrolled in the study after the identification of inclusion and exclusion criteria, including 12 patients with systemic JIA, 6 oligoarticular JIA, 8 RF-negative polyarticular JIA, 6 RF-positive polyarticular JIA, 8 ERA, 9 ReA, and 15 healthy controls. None of the patients was categorized as extended oligoarticular JIA. The median age at first visit for participants with JIA was 9.25 years (3.79, 11.50) and the median duration of disease was 2.38 months (2.00, 10.25). Thirty-one children (48.4%) were female, of whom one with oligoarthritis developed bilateral JIA-associated uveitis. Patients with JIA had a median number of 5.00 (3.00, 8.50) joints with active arthritis at the first visit, with a median score of 20.90 (15.35, 24.90), 34.15 (29.40, 39.25), and 5.0 (4.0, 5.5) in JADAS-27, sJADAS-27, and JSpADA, respectively. The positive rates of IL-4, IL-6, IL-10, TNF-α, IFN-γ, RF, and other autoantibodies at the first visit were 10.0, 45.0, 27.5, 15.0, 7.5, 12.5, and 34.38%, respectively. Twenty children (31.25%) with JIA in the cohort had a positive antinuclear antibody test. The baseline characteristics of JIA subgroups were listed in Table 1. Of note, levels of CRP, ESP, ferritin, and percentage of neutrophils were relatively increased in systemic JIA.

**Table 1:** Demographics and clinical characteristics of patients with JIA at baseline

	Systemic (*n* = 12)	Oligoarticular (*n* = 6)	RF-negative polyarticular (*n* = 8)	RF-positive polyarticular (*n* = 6)	Enthesitis-related arthritis (*n* = 8)
Age at first visit (years)	8.71 (3.92, 11.08)	2.29 (1.75, 4.67)	7.25 (3.46, 11.96)	9.63 (4.75, 13.25)	10.58 (9.38, 14.13)
Female	6 (50%)	4 (66.7%)	4 (50.0%)	5 (83.3%)	0 (0%)
Duration of disease (months)	2.00 (1.90, 2.08)	2.63 (2.30, 2.93)	5.80 (2.92, 13.18)	12.17 (2.17, 13.33)	1.85 (1.65,18.25)
Number of joints with active arthritis	5.50 (1.50, 13.00)	2.00 (1.00, 3.00)	5.50 (5.00, 7.50)	9.50 (7.00, 13.00)	4.00 (2.50, 5.00)
Percentage of neutrophils (%)	83.50 (71.50, 87.00)	51.50 (37.00, 58.00)	71.50 (58.50, 78.50)	65.00 (54.00, 72.00)	66.00 (63.50, 72.00)
C-reactive protein (mg/L)	59.00 (41.50, 84.00)	4.50 (4.00, 19.00)	24.5 (6.50, 47.50)	10.00 (7.00, 13.00)	22.00 (8.50, 31.00)
Erythrocyte sedimentation rate (mm/hour)	95.00 (73.00, 119.00)	20.00 (16.00, 24.00)	64.50 (58.50, 77.50)	58.50 (27.00, 82.00)	54.50 (38.50, 113.00)
Ferritin (ng/ml)	1220.45 (402.00, 3322.50)	43.65 (25.50, 58.10)	130.50 (65.85, 432.50)	36.85 (29.00, 53.00)	104.40 (53.95,199.00)

Data are median (IQR) and n (%) as appropriate. Abbreviations: *JIA* Juvenile Idiopathic Arthritis; *RF* Rheumatoid Factor

**Fig. 1:**
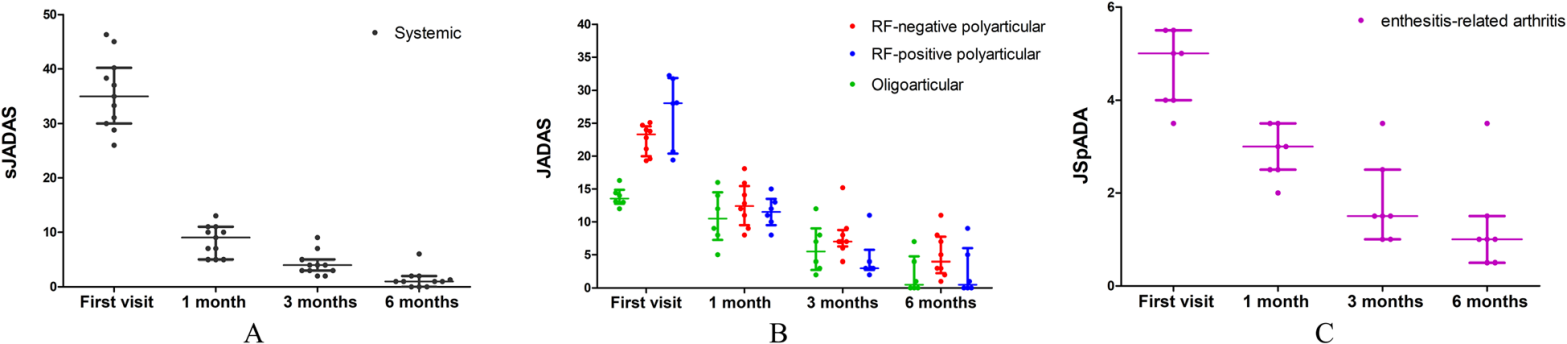
The disease activity score of JIA subgroups at each visit. The sJADAS (**A**), JADAS (**B**), and JSpADA (**C**) were decreased in JIA subgroups after individualized therapies. The scatter plot shows the median score as well as the 25th and 75th percentiles of the interquartile range. Abbreviations: sJADAS, systemic Juvenile Arthritis Disease Activity Score; JSpADA, Juvenile Spondyloarthritis Disease Activity Index; JADAS, Juvenile Arthritis Disease Activity Score; JIA, Juvenile Idiopathic Arthritis; RF, Rheumatoid Factor

In most patients with JIA, the quality of life was becoming more favorable and important inflammatory markers were also declining following 6-month personalized therapies, resulting in lower disease activity scores (Fig. 1). Twenty-three (71.9%) patients achieved minimal disease activity during the follow-up, of whom 69.6% reached the target of clinical remission. MRI-detected bone marrow edema was observed in six (15.0%) children and significantly associated with poor prognosis at the 6-month follow-up (*P* = 0.0143).

**Fig. 2:**
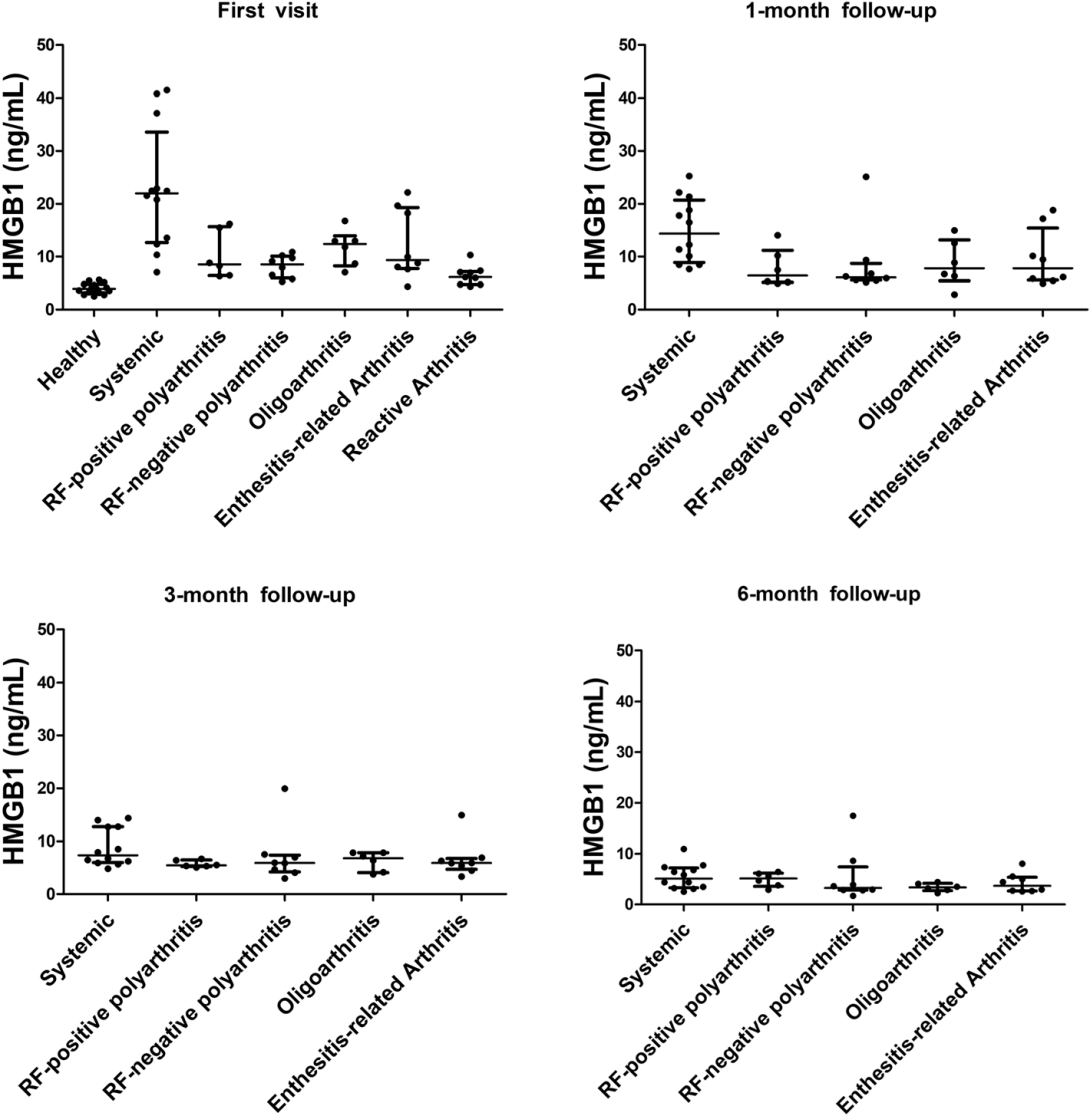
Levels of serum HMGB1 in different groups at each visit. The scatter plot shows the median level of serum HMGB1 as well as the 25th and 75th percentiles of the interquartile range. Abbreviations: HMGB1, high mobility group box 1 protein; RF, rheumatoid factor

At the onset of arthritis, levels of serum HMGB1 were significantly elevated in children with systemic JIA compared with healthy controls, the ReA group, and other JIA subgroups (*P* < 0.0001). Specifically, the median HMGB1 in systemic JIA was 21.98 (12.94, 30.00) ng/mL, as compared with 8.95 (7.35, 12.95) in non-systemic JIA (*P* = 0.0004). Meanwhile, patients with ERA had an increased level of HMGB1 as compared with healthy children (*P* = 0.0455). No significant differences were identified among other groups. Following 1-month therapies, HMGB1 levels among JIA subgroups were still of statistical significance (*P* = 0.0313), in which systemic JIA was the maximum. Serum HMGB1 levels were similar in all subgroups at the 3-month follow-up, and so was the 6-month follow-up. The HMGB1 levels of all groups at each visit were shown in Fig. 2.

**Fig. 3:**
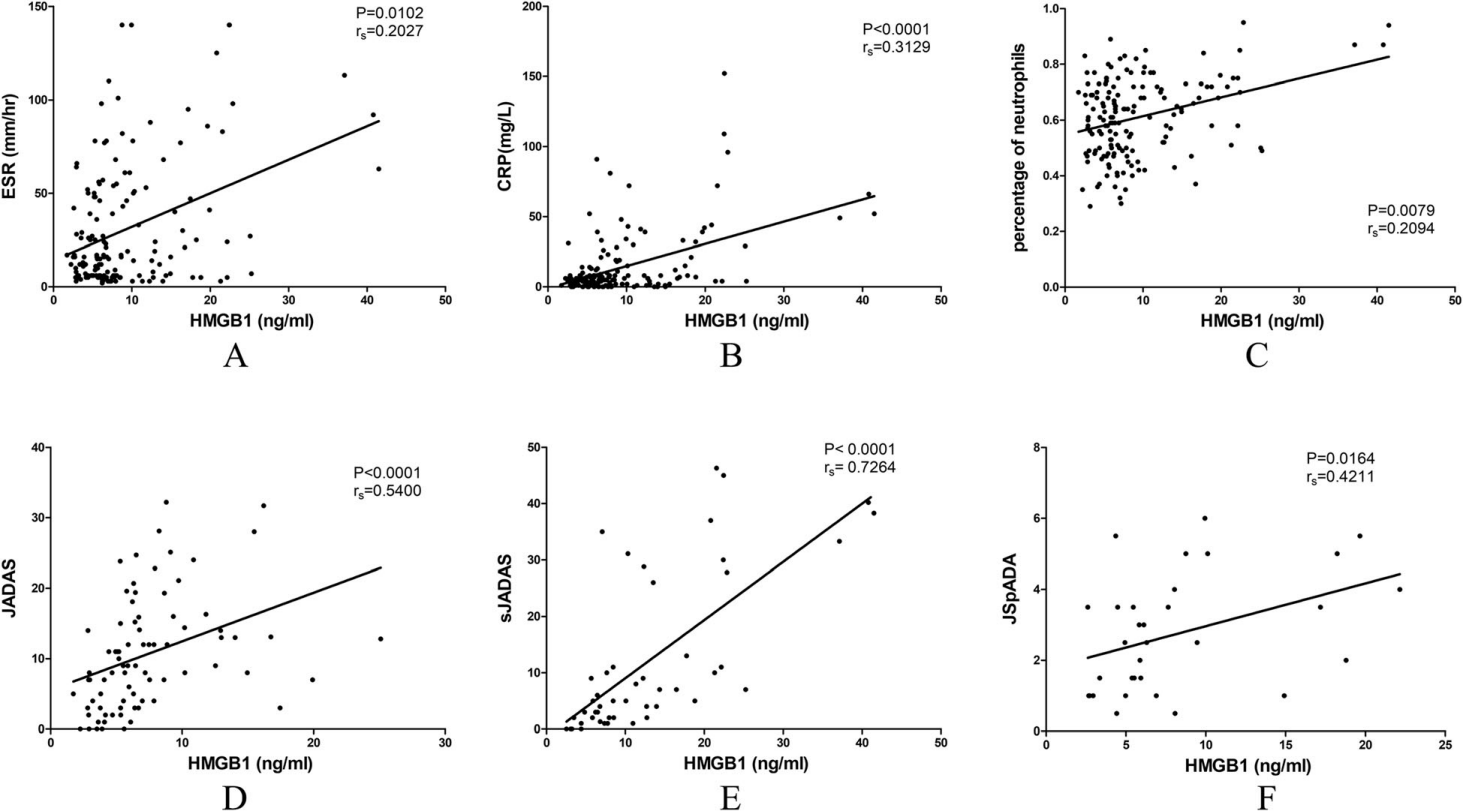
Correlations between HMGB1 and disease characteristics. ESR (**A**), CRP (**B**), percentage of neutrophils (**C**), JADAS-27 (**D**), sJADAS-27 (**E**), and JSpADA (**F**) were associated with HMGB1. The regression line, *P* values, and Spearman’s correlation coefficient (r_s_) are presented on the plot. Abbreviations: HMGB1, High Mobility Group Box 1 protein; ESR, Erythrocyte Sedimentation Rate; CRP, C-reactive protein; sJADAS, systemic Juvenile Arthritis Disease Activity Score; JSpADA, Juvenile Spondyloarthritis Disease Activity Index; JADAS, Juvenile Arthritis Disease Activity Score

Positive correlations were established at the first visit between HMGB1 levels and several inflammatory markers such as CRP (r_s_ = 0.4069, *P* = 0.0092), percentage of neutrophils (r_s_ = 0.3174, *P* = 0.0460) and ferritin (r_s_ = 0.3629, *P* = 0.0214). In the meantime, increased HMGB1 levels were associated with a shorter duration of disease (r_s_ = − 0.4604, *P* = 0.0028). During the 6-month follow-ups, levels of HMGB1 in different subgroups were not strongly correlated with disease activity or inflammatory markers at each visit. Nonetheless, data from all collected samples of patients with JIA revealed that serum HMGB1 levels were significantly associated with ESR (*P* = 0.0102), CRP (*P* < 0.0001), percentage of neutrophils (*P* = 0.0079), JADAS-27 (*P* < 0.0001), sJADAS-27 (*P* < 0.0001), and JSpADA (*P* = 0.0164), as illustrated in Fig. 3.

## Discussion

Apart from major pro-inflammatory cytokines such as IL-1β, IL-6, and TNF, HMGB1 plays a pivotal functional role in the pathogenesis of chronic inflammatory disorders and may operate as an alarmin both extracellularly and intracellularly [5]. Interestingly, it has been investigated that HMGB1 is likely to be involved in the development of autoimmune diseases such as systemic lupus erythematosus and rheumatoid arthritis [9, 11]. The identification of this biomarker in adults would integrate physicians’ interest with its function in JIA, which could shed further light on disease classification and new therapies [20, 21]. Recently, clinical features and inflammatory biomarkers have been evaluated in a Canadian cohort, indicating that their combination has improved the prediction of JIA outcomes [22, 23]. In this prospective longitudinal observational study, we aimed to analyze the levels of HMGB1 on different courses of JIA.

Consistent with previous knowledge, levels of serum HMGB1 were significantly higher in children with systemic JIA versus healthy controls and other types of arthritis in our study. Precisely speaking, the median levels in systemic JIA were almost increased six-fold than those in the control group. Systemic JIA has long been recognized as a unique type of pediatric chronic arthritis owing to its distinctive epidemiological and clinical features. Children with systemic arthritis do not demonstrate sex bias or peak age at onset and their extra-articular presentations, including fevers, macular rash, and serositis, can sometimes overshadow joint inflammation, appearing to be closely related to adult-onset Still’s disease [24]. Bobek et al. [25] indicated that children with systemic JIA had significantly higher serum levels of HMGB1 compared to the healthy controls, oligoarticular JIA, and polyarticular JIA. Another study reported that HMGB1 levels were significantly increased in synovial fluid versus blood samples, mainly in oligoarticular JIA [13]. Furthermore, levels of HMGB1 at the first visit were also significantly elevated in ERA compared with healthy controls in the present analysis, which has not been well documented so far. ERA is a category of JIA featured by back pain, chronic inflammatory arthritis, and enthesitis that involves inflammation at the insertion of a tendon, muscle, joint capsule, or ligament in the bone [26]. These children have an increased risk of developing inflammatory bowel disease and acute anterior uveitis, and almost 65–80% of them are positive for human leukocyte antigen-B27 that is strongly associated with spondyloarthritis. In adult patients with ankylosing spondylitis, serum HMGB1 was significantly higher and could reflect the disease activity [10]. Children with ERA may exhibit similar patterns in genetics and pathogenesis [27]. However, CRP is an important acute phase reactant in ERA and the absence of a significant correlation between CRP and HMBG1 is inconsistent with previous reports. The level of statistical significance was not robust to obtain a convincing association between HMBG1 and ERA, which may be attributable to small sample size. More evidence is required in the future to validate our findings.

The management of JIA remains difficult despite a growing number of effective drugs, as the clinical disease course is highly variable. Pediatricians need to make a comprehensive assessment at each visit to monitor the disease activity and optimize therapeutic strategies correspondingly. In general, the disease activity of JIA is measured by the JADAS in clinical practice with good results [16]. The sJADAS and JSpADA demonstrated good measurement properties as a valid assessment of disease activity in systemic JIA and ERA [18, 19]. Besides, biomarkers and imaging can provide useful information as well. Biomarkers are principally classified as biochemical (neutrophils, CRP, ESR), immunological (IL-6, TNF-α, IFN-γ), molecular, and genetic. Ultrasonography and MRI are also expected to become useful tools in detecting joint effusion and inflammation, especially bone marrow edema [28, 29]. However, clinically useful biomarkers with high sensitivity and specificity have not yet been recognized. In the present study, higher levels of HMGB1 at the first visit were associated with certain biomarkers and shorter disease duration, suggesting its function as an acute inflammatory reactant in JIA. Moreover, HMGB1 was related to the disease activity scores, especially sJADAS, based on the analysis of all samples from children with JIA, which indicated a specific marker for disease activity in JIA. Previous studies have demonstrated that HMGB1 levels were related to destructive JIA and more sensitive than CRP in detecting hepatosplenomegaly or serositis among patients with systemic JIA [14, 25]. Schierbeck et al. [13] found that HMGB1 in synovial fluid was the highest in patients with early disease onset irrespective of disease duration. Along with our findings, it has been supported that HMGB1 might be a pro-inflammatory mediator in the pathogenesis of JIA.

Our prospective analysis should be interpreted based on several noteworthy limitations: First of all, psoriatic JIA and undifferentiated JIA were not included because of their small percentages of patients with JIA at our institution. New classification criteria, slightly different from ILAR, have been developed to identify homogeneous chronic disorders that were historically termed with JIA [30]. Even now, controversy remains about the best approach for classifying chronic childhood arthritis [31–33]. Secondly, the patient population was relatively small with a short-term follow-up of 6 months, which restricts the power of our conclusions. Thirdly, the autoantibodies against HMGB1 were not examined in our cohort, and they have specific proteolytic activity against HMGB1 and interfere with the detection of HMGB1 using ELISA systems [20, 21, 34]. More efforts are needed to address these problems and determine its feasibility in the differential diagnosis and biological target therapy.

## Conclusions

In conclusion, serum HMGB1 may be associated with clinical disease activity of JIA and specifically increased at the first visit in children with systemic JIA, suggesting its function as a sensitive inflammatory marker and an immunotherapy target. It is possible to make an early diagnosis, monitor the disease activity, and define subgroups of JIA more precisely by measuring the serum level of HMGB1 in patients with systemic JIA or ERA. Further large-scale studies are warranted to explore its spectrum in JIA, making it clinically valuable and beneficial to the management of patients.

## Data Availability

Data are available on reasonable request. All data generated or analyzed during this study are included in this published article.

## Abbreviations

CRP: C-reactive protein
ELISA: Enzyme-linked immunosorbent assay
ERA: Enthesitis-related arthritis
ESR: Erythrocyte sedimentation rate
HMGB1: High mobility group box 1
IFN-γ: Interferon-γ
ILAR: International league of associations for rheumatology
IL: Interleukin
JADAS: Juvenile arthritis disease activity score
sJADAS: Systemic juvenile arthritis disease activity score
JSpADA: Juvenile spondyloarthritis disease activity index
JIA: Juvenile idiopathic arthritis
MRI: Magnetic resonance imaging
ReA: Reactive arthritis
RF: Rheumatoid factor
TNF-α: Tumor necrosis factor-α
